# Exploring Personal and Relational Competencies for Enhancing Nursing Performance: Focusing on Communication Skills, Grit, and Leader–Member Exchange

**DOI:** 10.3390/nursrep16060207

**Published:** 2026-06-22

**Authors:** Hyunmin Lee, Sukjae Park, Eunhee Shin

**Affiliations:** 1 Samsan Hospital, 5, Hyeoksin-ro, Wonju-si 26411, Republic of Korea; gusals705@naver.com; 2 Kangbuk Samsung Medical Center, Seoul 03181, Republic of Korea; psj930925@naver.com; 3 Department of Nursing Science, College of Health Sciences, Sangji University, Wonju 26339, Republic of Korea

**Keywords:** nursing performance, communication skills, grit, leader–member exchange (LMX), clinical nurse

## Abstract

**Background**: This study examined how clinical nurses’ communication competence, grit, and leader–member exchange (LMX) contribute to nursing performance, aiming to identify key predictors to support workforce development and organizational policy planning. **Methods**: This study was conducted as a descriptive correlation study targeting 190 clinical nurses working at a general hospital in S City. Data were collected through a self-report questionnaire and analyzed using descriptive statistics, *t*-test, ANOVA, Pearson correlation coefficient, and hierarchical regression analysis using the IBM SPSS/WIN 25.0 program. **Results**: On a 5-point scale, the mean score of nursing performance was 4.05. Communication skills (β = 0.52, *p* < 0.001), grit (β = 0.23, *p* = 0.002), and clinical experience (β = 0.18, *p* = 0.022) significantly affected nursing work performance, with communication skills having the greatest effect. This model explained 47.3% of the nursing performance. **Conclusions**: To improve the nursing performance of nurses, strengthening communication skills and grit, supporting professional development, and recognizing clinical experience are crucial. These findings suggest that integrating such programs into ward nurse training may contribute to improved nursing performance and organizational effectiveness. To develop these competencies and evaluate their effectiveness, follow-up research is continuously needed.

## 1. Introduction

Patients increasingly seek hospitals that provide high-quality healthcare services, and hospitals strive to improve the quality of care to enhance patient satisfaction and competitiveness [1,2]. Nurses, who are the primary healthcare professionals directly interacting with patients, play a critical role in delivering high-quality nursing care and influencing patient satisfaction [3,4]. Therefore, hospitals are making significant efforts to improve nurses’ work performance and the overall quality of nursing services [5].

Improved nursing performance contributes to the delivery of safe, effective, and high-quality patient care through the appropriate application of the nursing process [6].

To improve the performance of nursing organizations, several studies have been conducted to identify factors influencing nursing performance [5,7]; personal, organizational, and environmental factors influence nursing performance [8]. Previous studies have reported that factors associated with improving nursing work performance encompass communication ability and job placement [9], the leader–member exchange (LMX) relationship and organizational commitment [10], self-leadership [11], resilience [12,13], grit [11], nursing competency, and job satisfaction [14]. Leader–member exchange refers to the quality of the relationship between supervisors and employees, characterized by mutual trust, respect, support, and reciprocal interactions within the workplace. In nursing settings, LMX reflects the quality of the relationship between nurse managers and staff nurses and may influence communication, psychological support, work motivation, and organizational commitment. High-quality LMX can facilitate supportive work environments and collaborative interactions, which may ultimately contribute to improved nursing performance.

Among these related factors, higher levels of individual competencies are associated with better performance outcomes [15]. When nurses have high job satisfaction or organizational commitment, they are more likely to pursue organizational goals and effectively utilize their skills to provide high-quality patient care, thereby improving nursing performance [5].

Previous studies have reported that higher communication competence is associated with increased job satisfaction among nurses [16], whereas higher levels of grit are related to greater job satisfaction [17]. In addition, high-quality leader–member exchange (LMX) has been associated with stronger organizational commitment [18]. Furthermore, organizational commitment and job satisfaction have been reported as important factors related to improved nursing performance [4,10].

However, although communication competence, grit, and LMX have each been associated with positive nursing outcomes, most previous studies have examined these variables independently. Limited research has investigated their integrated influence on nursing performance among hospital nurses. Furthermore, insufficient attention has been given to how interpersonal competencies, psychological resources, and relational workplace factors collectively contribute to nursing performance in complex clinical settings.

Nurses face increasing workload, emotional demands, and complex care environments that may hinder optimal performance. In such conditions, interpersonal and psychological competencies—including communication competence, grit, and high-quality LMX—may play an essential role in enhancing performance, teamwork, and sustained engagement. Despite growing interest, most studies have examined communication competence, grit, or LMX separately, rather than within an integrated framework predicting nursing performance.

Therefore, this study aimed to provide empirical evidence on effective nursing organization operation and nursing performance in hospitals by identifying the correlation between communication skills, grit, LMX, and nursing performance with each variable and analyzing the effect of each variable on clinical nursing performance among hospital nurses.

The specific objectives were to:

(1) Assess the levels of communication competence, grit, LMX, and nursing performance;
(2) Examine the relationships among these variables;
(3) Identify the factors associated with nursing performance.

This study was grounded in Social Exchange Theory, which posits that high-quality relationships between employees and supervisors promote reciprocal trust, support, and positive work-related outcomes. Within this framework, leader–member exchange (LMX) reflects the quality of the relational environment in which nurses perform their work and may influence motivation, organizational commitment, and work performance.

Communication competence refers to the ability to effectively exchange information, collaborate with others, and respond appropriately in clinical situations, all of which are essential for safe and high-quality nursing care. Grit, defined as perseverance and sustained passion toward long-term goals, may help nurses maintain engagement and resilience despite stressful clinical environments.

Accordingly, communication competence and grit were conceptualized as individual and psychological resources that directly contribute to nursing performance, whereas LMX was considered a relational organizational factor that may support or strengthen these associations.

## 2. Methods

### 2.1. Study Design and Setting

This study used a quantitative, cross-sectional descriptive correlational design to examine the relationships among communication skills, grit, leader–member exchange (LMX), and nursing performance among clinical nurses. Participants were recruited using convenience sampling from a general hospital located in S City. Eligible participants were registered nurses with at least 6 months of clinical experience; nurses working in specialized departments such as pediatrics, the operating room, intensive care units, and neonatal care units were excluded. Participation was voluntary, informed consent was obtained from all participants, and the study was approved by the Institutional Review Board (IRB).

### 2.2. Participants

This study was conducted among clinical nurses, and those working in specialized departments were excluded because their communication patterns differ from those of ward nurses [19]. For example, intensive care unit and operating room nurses have limited direct interaction with conscious patients and typically work in specialized teams [19]. Therefore, nurses in specialized departments were excluded because their interactions with patients, fellow nurses, and physicians differ from those of ward nurses.

The sample size was determined using the G*Power 3.1.9.7 program with α = 0.05, power = 0.90, effect size f = 0.15, and 10 predictors, resulting in a required minimum sample size of 172 participants. Participants were recruited using convenience sampling from a tertiary general hospital located in S City, Republic of Korea. Data were collected from 14 August to 23 August 2024. Considering a dropout rate of 25%, a total of 230 questionnaires were distributed. Of these, 210 were returned, yielding a response rate of 91.3%. After excluding 20 incomplete or invalid responses, data from 190 participants were included in the final analysis.

### 2.3. Sampling Method

Participants were recruited using convenience sampling from a tertiary general hospital located in S City, Republic of Korea. Data were collected from 14 August to 23 August 2024. After obtaining approval from the hospital nursing department, recruitment notices describing the study purpose and procedures were posted in each ward with the cooperation of unit managers. Nurses who voluntarily agreed to participate after reading the study information sheet provided written informed consent and completed the questionnaire. Completed questionnaires were submitted in designated sealed collection envelopes, and a research assistant subsequently collected the questionnaires from each department.

### 2.4. Ethical Consideration

This study received approval from the IRB of S City General Hospital where the survey was conducted (approval number: KBSMC 2024-06-035-003). Eligible participants were recruited through notices posted in each ward. Nurses who agreed to participate provided written informed consent after receiving an explanation of the study purpose, procedures, potential inconvenience, confidentiality, and data protection measures. Participants were informed that they could withdraw from the study at any time without any disadvantage. All questionnaires were collected anonymously and stored securely to ensure participant confidentiality. All study procedures were conducted in accordance with the principles of the Declaration of Helsinki.

### 2.5. Measurement

#### 2.5.1. Nursing Performance

Nursing performance was measured using the Korean nursing performance measurement tool developed by Ko et al. [8]. The instrument consists of 17 items across four subdomains: nursing competency (7 items), nursing attitude (4 items), improvement in nursing practice (3 items), and application of the nursing process (3 items). Each item was rated on a 5-point Likert scale ranging from 1 (“not at all”) to 5 (“very much”), with higher scores indicating higher nursing performance. Cronbach’s α was 0.92 in the study by Ko et al. [8] and 0.95 in the present study.

#### 2.5.2. Communication Skills

Communication skills were measured using the Korean version of the Global Interpersonal Communication Competence Scale originally developed by Heo [20] and later modified for healthcare professionals by Lee and Kim [21]. The instrument consists of 15 items assessing empathy, assertiveness, social appropriateness, interaction management, and coherence. Each item was rated on a 5-point Likert scale, with higher scores indicating better communication skills. Items 10 and 11 were reverse-scored. Cronbach’s α was 0.72 in the original development study, 0.83 in the study by Lee and Kim [21], and 0.87 in the present study.

#### 2.5.3. Grit

Grit was measured using the Korean grit scale developed by Park et al. [22]. The instrument comprises 14 items across three domains: perseverance for long-term goals (5 items), passion for the profession (5 items), and patient-oriented intrinsic motivation (4 items). Each item was rated on a 5-point Likert scale, with higher scores indicating higher grit. Cronbach’s α was 0.91 in the original study and 0.92 in the present study.

#### 2.5.4. LMX

Leader–member exchange (LMX) was measured using the Korean version of the Multi-Dimensional Measure of LMX originally developed by Liden and Maslyn [23] and adapted for nurses by Kim and Jeon [24]. The instrument consists of 11 items across four subdomains: affect (3 items), loyalty (3 items), contribution (2 items), and professional respect (3 items). Each item was rated on a 5-point Likert scale, with higher scores indicating higher-quality LMX relationships. Cronbach’s α was 0.91 in the study by Kim and Jeon [24] and 0.95 in the present study.

### 2.6. Statistical Methods

The data collected in this study were analyzed using IBM SPSS/WIN 25.0. Descriptive statistics, including means, standard deviations, minimum values, and maximum values, were calculated for communication skills, grit, leader–member exchange (LMX), and nursing performance. Prior to conducting parametric analyses, the assumptions of normality were assessed, and all variables satisfied the criteria for normal distribution. Independent t-tests, one-way analysis of variance (ANOVA), and Scheffé post hoc tests were conducted to examine differences in nursing performance according to participants’ characteristics. Pearson’s correlation coefficients were used to examine relationships among communication skills, grit, LMX, and nursing performance. Hierarchical regression analysis was performed to identify factors influencing nursing performance. The assumptions of regression analysis, including linearity, normality, homoscedasticity, independence of residuals, and multicollinearity, were examined and satisfied. The Durbin–Watson statistic was 2.144, indicating no autocorrelation. Tolerance values ranged from 0.443 to 0.973, and variance inflation factor (VIF) values ranged from 1.028 to 2.255, indicating no multicollinearity. Statistical significance was set at *p* < 0.05.

## 3. Results

### 3.1. General Characteristics of the Participants

The general characteristics of the participants are presented in Table 1. A total of 190 nurses participated, most of whom were female (97.4%). Most participants were aged 23–30 years. Nearly all participants held a bachelor’s degree (96.3%). Most were single (79.5%). Over half reported no religious affiliation.

Clinical experience averaged 66.54 months (SD = 56.47), and 41.6% had more than five years of experience. More than half worked in internal medicine departments (54.7%), followed by surgical units (40.5%).

Most participants earned between 3.0 and 3.49 million Korean won (KRW) per month. Most participants reported moderate to high levels of job and departmental satisfaction.

Detailed characteristics are provided in Table 1.

### 3.2. Participants’ Communication Skills, Grit, LMX Relationship, and Nursing Performance

The mean scores for communication skills, grit, LMX, and nursing performance ranged from 3.70 to 4.05 on a 5-point scale, indicating generally moderate to high levels among participants. Nursing performance demonstrated the highest mean score, followed by communication skills, LMX, and grit. As no substantial deviation from normality was identified, parametric statistical analysis was considered appropriate (Table 2).

### 3.3. Differences in Communication Skills, Grit, and LMX According to Participants’ General Characteristics

The differences between communication skills, grit, LMX relationship, and nursing performance according to participants’ general characteristics are presented in Table 3. Communication skills exhibited statistically significant differences in terms of department satisfaction (F = 5.06, *p* = 0.007) and job satisfaction (F = 7.77, *p* = 0.001). Post-test results showed that department satisfaction was higher when satisfied than dissatisfied, and job satisfaction was higher satisfied than average and dissatisfied. Grit showed statistically significant differences in terms of average salary (F = 5.58, *p* = 0.004), department satisfaction (F = 7.74, *p* = 0.001), and job satisfaction (F = 15.11, *p* < 0.001). As a result of the post-test, Nurses with a monthly salary of ≥4 million won demonstrated significantly higher communication skills, grit, LMX, and nursing performance than those with lower salary levels, and department satisfaction and job satisfaction had more counts of satisfactory than average or dissatisfied. There were statistically significant differences in department (F = 3.14, *p* = 0.046), compensation satisfaction (F = 6.32, *p* = 0.002), department satisfaction (F = 23.32, *p* < 0.001), and job satisfaction (F = 16.28, *p* < 0.001) related to LMX. The other departments were higher than the internal medicine department, and the compensation satisfaction, department satisfaction, and job satisfaction had higher counts of satisfied than dissatisfied. Regarding nursing performance, statistically significant differences were observed in age (F = 6.35, *p* = 0.002), marital status (t = −2.93, *p* = 0.004), total clinical experience (F = 5.36, *p* < 0.001), average compensation (F = 4.20, *p* = 0.016), and job satisfaction (F = 3.80, *p* = 0.024). The 31–40-year age group had significantly higher communication skills scores than the 23–30-year age group. Grit and LMX were significantly higher among married nurses than among unmarried nurses, and when total clinical experience was more than 5 years, average salary was more than 4 million won, job satisfaction was satisfactory, and nursing job performance was high.

### 3.4. Correlation Between Communication Skills, Grit, LMX, and Nursing Performance

In this study, nursing performance was statistically significantly positively correlated with communication skills (r = 0.587, *p* < 0.001), grit (r = 0.499, *p* < 0.001), and LMX relationship (r = 0.200, *p* < 0.001), with communication skills exhibiting the highest positive correlation (Table 4).

### 3.5. Nursing Performance-Associated Factors

Before conducting a regression analysis to identify the factors affecting nursing performance, normality, homoscedasticity, and independence of residuals were examined to verify the suitability of the regression analysis model. The assumptions of homoscedasticity, normality, and independence were satisfied, and the regression model of this study was suitable. The Durbin–Watson value was 2.144, which is between the standard values of 1 and 3; therefore, it was confirmed to be independent without autocorrelation. The tolerance limit was approximately 0.443–0.973, which is 0.1 or more, and the variance expansion factor was approximately 1.028–2.255, which is <10, indicating no problem with multicollinearity.

To identify factors affecting the participants’ nursing performance, nursing performance was used as the dependent variable, and hierarchical multiple regression analysis was conducted by entering variables, including age, marriage, clinical experience, average salary, and job satisfaction, showing significant differences in the nursing performance level according to the general characteristics of the participants, and communication skills, grit, and LMX relationship, which showed significant correlations with nursing performance. Of these, five variables, including age, marriage, clinical experience, average salary, and job satisfaction, were treated as dummy variables and entered as independent variables.

To verify the factors affecting the participants’ nursing performance in stages, the general characteristics affecting nursing performance, LMX relationship, grit, and communication skills were entered into Models I, II, III, and IV, respectively, and a hierarchical regression analysis was conducted in four stages. In Model I, general characteristics were entered to control for potential confounding effects. Communication competence was entered in Model II because it represents a core professional competency directly related to nursing practice. Grit was added in Model III as an individual psychological resource, and LMX was entered in Model IV to examine whether relational factors explained additional variance in nursing performance beyond personal competencies. Finally, the results of the study in Model IV revealed that communication skills (β = 0.52, *p* < 0.001), grit (β = 0.23, *p* = 0.002), and clinical experience (>5 years) (β = 0.18, *p* = 0.022) was significantly associated with nursing performance in that order. Model IV demonstrated a 47.3% explanatory power, which was 16.8% higher than that of Model III, and the F value of the regression model fit was 22.20 (*p* < 0.001). Higher communication, higher grit, and >5 years of clinical experience were associated with higher nursing performance (Table 5).

The order of variable entry in the hierarchical regression analysis was theoretically driven. General characteristics that showed significant differences in nursing performance were entered first to control for their potential confounding effects. Communication competency was entered next because it represents a core professional competency directly related to nursing practice. Grit was then added as an individual psychological resource. Finally, LMX was entered to examine whether relational factors explained additional variance in nursing performance beyond personal competencies.

## 4. Discussion

The differences in nursing performance according to the general characteristics of the study participants indicated that nurses aged 31–40 years, married, had >5 years of total clinical experience, had a monthly salary of >4 million won, and had high job satisfaction exhibited higher nursing performance. These results are comparable to and consistent with those reported in previous studies demonstrating differences in nursing performance according to age, marital status, work experience, and salary level [9,21,24,25].

Regarding age-related characteristics, nursing performance was higher when the age was 31–40 years, which is consistent with the study by Lim [25] targeting nurses in small- and medium-sized hospitals and the study by Son et al. [19] targeting nurses in university hospitals. Studies by Lee & Yeom [26] and Lim [25] reported that nursing performance increases as clinical experience accumulates, which is consistent with the findings of the present study. This finding may reflect the synergistic effects of increasing age and clinical experience on nursing performance [27], as well as greater confidence and professional expertise acquired through practice [19]. Therefore, to prevent career nurses with extensive clinical experience from changing jobs, organizational support, such as considering each nurse’s personal circumstances and assigning them to departments that match their aptitude, may help improve their nursing performance.

Regarding marital status, married nurses demonstrated better nursing performance than unmarried ones. This result is consistent with those of Kwon et al. [28] and Cheon [9]. A possible explanation is that marriage offers social and economic stability, and this stability may positively influence job performance. Moreover, the higher the average salary, the higher the nursing performance, which agrees with the findings of Kwon et al. [28], Kim [29], Lee & Yeom [26], and Cheon [9]. As appropriate economic compensation is associated with enhanced nursing performance, improving the compensation system according to the clinical experience, nursing performance, and ability of nurses may be beneficial.

In this study, the average score of clinical nurses’ communication skills, the variable with the greatest influence on nursing performance, was 3.87 ± 0.41 of 5 points. In the study by Lee & Kim [21], which employed the same tool for hospital nurses, the average score was 3.46 points, which was lower than this study. Furthermore, in the study by Cheon [9], which used a different tool for nurses in small- and medium-sized hospitals, the average score was 3.48 points. In the study by Kim [29] that included outpatient nurses, it was 3.34 points, which was lower than this study. Previous studies that evaluated the communication skills of nurses have reported slight differences in the communication skill scores depending on the study participants. Variations in communication competence scores across studies may reflect differences in clinical settings, organizational cultures, and participant characteristics. In the present study, ward nurses demonstrated relatively high communication competence, possibly because they frequently engage in direct patient interaction, interdisciplinary collaboration, and care coordination in dynamic clinical environments [9,20,29,30]. The participants of the present study were clinical nurses, excluding outpatient nurses, ward managers, insurance review nurses, and nurses in specialized departments (pediatrics, operating rooms, intensive care units, neonatal units, etc.) in medical institutions. It was believed that ward nurses had higher communication skills scores because they had more opportunities to communicate than those in specialized departments and managers. Nurses constantly interact with patients and their guardians in the process of performing nursing work, and communication skills are essential when collaborating with members of other departments in the hospital, resolving work coordination situations, and handing over work between nurses. Therefore, improving communication skills is considered an essential element in enhancing nursing performance. Implementing programs that help nurses develop their communication skills is necessary, and it is believed that strengthening and implementing communication education and training programs that particularly develop the ability to logically assert one’s position is essential.

In this study, the grit score of the participants was 3.71 ± 0.50. Although not using the same tool, the study by Jeong [31] measuring grit for university hospital nurses reported an average grit score of 3.12, comparable to the result of this study. The study by Lee [32] targeting nurses in small- and medium-sized hospitals reported an average grit score of 2.91, which was lower than the result of this study. These results suggest that the level of grit of nurses can vary depending on the hospital size. In this study, the scores of the sub-areas of grit were the highest for “passion to become a nursing professional” at 3.93 ± 0.48, followed by “patient-oriented intrinsic motivation” at 3.86 ± 0.58 and “persistence to achieve long-term goals” at 3.32 ± 0.69. This finding indicates that although clinical nurses have a high passion for pursuing nursing professionalism, they are relatively lacking in the persistence to maintain it. The study by Lee [33] also emphasized the lack of persistence among nurses, reporting that nurses frequently change jobs or resign owing to conflicts arising from the disparity between high practical expectations and reality. Moreover, higher organizational commitment and job satisfaction led to higher nursing work performance [4,10]. This may be associated with the lack of persistence for long-term goals. Perseverance, endurance, and continuous and voluntary self-growth abilities are highly crucial for nurses [33]. In particular, grit, which includes the concepts of perseverance, passion, and intrinsic motivation, plays a significant role in meeting the requirements of nurses as professionals who must solve patients’ problems within a limited time, continuously learn, and grow owing to the nature of their job that deals with life. Therefore, nurses with high grit levels may exhibit higher work performance. Accordingly, developing support and education programs that foster nurses’ grit at the hospital organization level is necessary, and establishing an organizational culture and work environment that allows nurses to demonstrate grit is essential. Moreover, each clinical nurse must maintain their passion and make conscious and consistent efforts to achieve their goals.

The mean LMX score was 3.78 ± 0.65, with intellectual respect being the highest subscale. Although previous studies have reported similar patterns in LMX dimensions [18,34,35], the correlation between LMX and nursing performance in this study was relatively weak (r = 0.200). This suggests that while LMX is statistically significant, its practical contribution to nursing performance appears modest compared to stronger predictors such as communication skills and grit. One possible explanation is that nursing performance in clinical settings may depend more directly on individual competencies and psychological resources than on supervisory relationships.

The hierarchical and task-oriented structure of Korean nursing organizations may also limit the development of high-quality LMX relationships. Given that decision-making authority is often centralized and interactions with supervisors are formal and directive, opportunities for individualized leader–member exchange may be restricted, particularly for early-career nurses. Previous research similarly highlights the influence of cultural norms such as seniority and organizational hierarchy in shaping relational dynamics within healthcare teams [36].

Furthermore, previous studies have reported that high-quality LMX relationships contribute to positive organizational outcomes, including organizational commitment and customer-oriented behaviors, supporting the theoretical importance of LMX in workplace settings [36,37].

Although leader–member exchange (LMX) was significantly correlated with nursing performance in the bivariate analysis, its effect was no longer statistically significant after communication competence and grit were entered into the final regression model. This finding suggests that the association between LMX and nursing performance may be partially explained by individual competencies and psychological resources. In particular, nurses with higher communication competence and greater grit may be more likely to establish positive relationships with their supervisors, thereby reducing the independent contribution of LMX to nursing performance. These results indicate that, although relational factors such as LMX are important in the nursing work environment, individual professional competence and psychological resources may play a more direct role in predicting nursing performance among clinical nurses.

Therefore, the nonsignificant effect of LMX in the final model likely reflects contextual and structural characteristics of the nursing work environment rather than a lack of theoretical importance. Future studies should explore moderating variables—including leadership style, communication climate, and staffing culture—and investigate whether LMX exerts stronger effects in decentralized or collaborative care environments. While LMX may still play a role in enhancing teamwork and psychological safety, its direct impact on measurable performance outcomes appears limited. Additionally, as this study utilized a cross-sectional design, causal interpretations should be avoided. Longitudinal or intervention-based research is needed to better understand how LMX evolves over time and how relational patterns may influence performance in different clinical settings.

Nursing performance was positively correlated with communication skills, grit, and LMX, which is consistent with previous studies [11,19,30,33]. In particular, communication skills showed the highest correlation with nursing performance, aligning with the study by Cheon [9].

As a result of the hierarchical regression analysis, the explanatory power of the general characteristics in Model I was 9%, after including LMX in Model II it rose to 10%, suggesting a minimal contribution. When grit was added in Model III, the explanatory power increased to 30.5%, and when communication skills were added in Model IV, variance explained increased substantially to 47.3%. Therefore, communication skills and grit were confirmed to be the main factors associated with higher nursing performance; clinical experience was also an influential general factor. Among the general characteristics, total clinical experience was demonstrated to be an influential factor.

Communication competence emerged as the strongest predictor of nursing performance, consistent with the findings of Son et al. [19]. This may be because nursing practice relies heavily on accurate information exchange, interdisciplinary collaboration, patient education, and rapid clinical decision-making. Nurses with higher communication competence may be better able to coordinate care, reduce misunderstandings, and respond effectively to complex clinical situations. Because nurses frequently interact with patients, family members, and healthcare professionals, effective communication contributes to trust-building, smoother collaboration, and improved patient experiences. Even during routine nursing care, nurses with strong communication competence may help patients and their families feel more comfortable and understood, thereby enhancing the perceived quality of care. Therefore, improving nurses’ communication competence may lead to better nursing performance. Structured communication training and continuing education programs should be implemented to strengthen nurses’ ability to communicate clearly, confidently, and effectively in clinical settings.

Grit was the second most influential factor associated with nursing performance, and previous studies have demonstrated that grit positively influences nursing performance [32,38]. Previous research has also shown that nursing performance is higher when nurses perceive a supportive work environment, positive relationships with colleagues within and across departments, and greater awareness of the disadvantages associated with turnover [38]. Moreover, the findings of previous studies were consistent with those of the present study, indicating that grit is an influential factor in increasing job satisfaction [39]. In the present study, grit was significantly associated with nursing performance, suggesting that sustained effort and perseverance are important for maintaining work performance in demanding clinical environments. In busy and emotionally challenging settings, nurses with greater grit may be more likely to persist despite fatigue, workload, and interpersonal stress.

Therefore, nursing managers should help nurses develop their grit by assigning tasks that fit their characteristics and by creating a work environment that promotes positive interpersonal relationships and sustained motivation. Furthermore, nurses should be provided with opportunities for promotion. Offering educational opportunities for skill development and self-improvement, as well as appropriate compensation according to nurses’ abilities and clinical experience, may strengthen both intrinsic and extrinsic motivation, thereby improving nursing performance. Accordingly, programs to improve and strengthen nurses’ grit should be developed at the organizational level.

Among the general characteristics of the study participants, total clinical experience was a factor affecting nursing performance. This finding is consistent with those of Kwon [40], who reported a significant difference in nursing performance depending on the clinical experience of nurses in intensive care units. The competency of experienced nurses accumulated through abundant knowledge and clinical experience in patient nursing work can be identified as a factor that significantly affects nursing performance. Moreover, based on the results of previous studies indicating that clinical experience showed a significant difference, clinical experience can be confirmed as a major factor in nursing performance. In this study, total clinical experience was confirmed as a key factor; however, as previous studies have also investigated the intensive care unit work experience, it is believed that further studies encompassing various samples will be necessary. Future studies including diverse clinical environments are recommended.

To improve the communication competence and strengthen the grit of clinical nurses, a supportive nursing organizational culture and positive work environment should be established. In particular, nursing performance may be enhanced through educational programs that strengthen nurses’ communication competence and grit, evaluation of individual suitability for specific roles and departments, and organizational support that promotes positive interpersonal relationships and professional development. In addition, providing appropriate compensation and welfare benefits may contribute to improving nursing performance and reducing turnover. These findings contribute to the growing body of evidence suggesting that it provides foundational evidence for developing policies and intervention strategies to enhance the nursing performance of clinical nurses and prevent turnover. Based on these findings, hospitals should consider implementing structured communication training programs, such as simulation-based communication workshops and interdisciplinary team communication exercises. Structured communication training programs, interdisciplinary simulation exercises, and reflective practice interventions may help strengthen communication competence among clinical nurses. In addition, organizational strategies aimed at enhancing grit—such as mentoring systems, resilience-building programs, supportive leadership, and professional development opportunities—may contribute to improved nursing performance and retention.

Programs designed to strengthen grit, resilience, and sustained motivation may also be beneficial. For example, mentoring programs, reflective practice sessions, and resilience-building workshops could help nurses maintain performance in demanding clinical environments.

This study has several limitations. First, because a cross-sectional design was used, causal relationships cannot be established. Second, the study was conducted at a single hospital using convenience sampling, which may limit the generalizability of the findings and introduce selection bias. Third, all variables were measured using self-report questionnaires, which may have introduced response bias, social desirability bias, and common method variance. In addition, advanced practice nurses were excluded; therefore, the findings may not be applicable to nurses in specialized roles. Finally, potential confounding variables such as workload, burnout, staffing levels, and organizational climate were not controlled for. Future studies should include these variables and employ longitudinal or multi-site designs.

## 5. Conclusions

This study showed that higher levels of communication competence and grit were associated with higher clinical nursing performance. Among these factors, communication competence emerged as the strongest predictor, followed by grit and longer clinical experience. Although leader–member exchange was positively correlated with nursing performance, its independent contribution was limited after communication competence and grit were considered. Performance was also higher among nurses with greater clinical experience, higher salaries, and higher job satisfaction.

Nursing performance may be enhanced when strategies focus on strengthening individual competencies and improving organizational working conditions. Practically, hospitals should integrate structured communication training and grit-building interventions into mandatory clinical education programs for ward nurses. Furthermore, policies supporting nurse retention—such as competitive salary structures, workload management, and clear career-development pathways—may help facilitate the accumulation of clinical experience and sustained engagement. Establishing standardized continuing education requirements and leadership support systems at the institutional or national level may also contribute to improving nursing performance and care quality.

However, because data were collected from a single hospital using non-probability sampling, the generalizability of these findings may be limited. Future multi-site and longitudinal studies are needed to validate these associations and determine whether targeted educational or policy-level interventions lead to measurable improvements in nursing performance.

## Figures and Tables

**Table 1:** General characteristics of the participants (N = 190).

Variables	Categories	N (%)
Sex	Male	5 (2.4)
	Female	185 (97.4)
Age (years)	23–30	131 (68.9)
	31–40	50 (26.3)
	41–50	9 (4.7)
Education level	Bachelor’s degree	183 (96.3)
	Master’s degree or higher	7 (3.7)
Marital status	None	151 (79.5)
	Yes	39 (20.5)
Religion	Yes	70 (36.8)
	No	120 (63.2)
Clinical experience (years)	Average	66.54 ± 56.47
	From approximately 6 m to <1 year	11 (5.8)
	From approximately 1 to <3	59 (31.1)
	From approximately 3 to <5	41 (21.6)
	≥5	79 (41.6)
Working department	Medical ward	104 (54.7)
	Surgical ward	77 (40.5)
	Other departments	9 (4.7)
Monthly income (1000 won)	<2990	25 (13.2)
	Approximately 3000–3990	147 (77.4)
	≥4000	18 (9.5)
Salary satisfaction	Satisfied	30 (15.8)
	Neutral	91 (47.9)
	Dissatisfied	69 (36.3)
Department satisfaction	Satisfied	107 (56.3)
	Neutral	64 (33.7)
	Dissatisfied	19 (10)
Job satisfaction	Satisfied	81 (42.6)
	Neutral	85 (44.7)
	Dissatisfied	24 (12.6)

**Table 2:** Participants’ communication skills, grit, leader–member exchange (LMX) relationship, and nursing performance (N = 190).

Variables	Range	M ± SD	Min	Max	Skewness	Kurtosis
Communication skills	1–5	3.87 ± 0.41	2.67	5.0	−0.037	0.587
Grit	1–5	3.70 ± 0.50	2.36	5.0	0.080	0.376
LMX	1–5	3.78 ± 0.65	1.45	5.0	−0.410	0.792
Nursing performance	1–5	4.05 ± 0.44	3.00	5.0	0.224	0.016

**Table 3:** Differences in communication skills, grit, and LMX according to participants’ general characteristics (N = 190).

Variables	Categories	Communication Skills		Grit		LMX		Nursing Performance	
		M ± SD	t or F (*p*) Scheffe	M ± SD	t or F (*p*) Scheffe	M ± SD	t or F (*p*) Scheffe	M ± SD	t or F (*p*) Scheffe
Sex	Male	3.88 ± 0.40	0.32 (0.765)	3.69 ± 0.50	−0.69 (0.491)	3.77 ± 0.64	−1.66 (0.098)	4.05 ± 0.44	−0.02 (0.984)
	Female	3.77 ± 0.72		3.84 ± 0.25		4.25 ± 0.55		4.06 ± 0.47	
Age (years)	23–30	3.90 ± 0.41	0.77 (0.464)	3.68 ± 0.50	0.41 (0.665)	3.77 ± 0.67	0.49 (0.614)	3.98 ± 0.41	6.35 (0.002) a < b
	31–40 ^b^	3.82 ± 0.40		3.70 ± 0.50		3.85 ± 0.63		4.23 ± 0.44	
	41–50 ^c^	3.83 ± 0.43		3.83 ± 0.48		3.66 ± 0.38		4.15 ± 0.56	
Education level	Bachelor’s degree	3.88 ± 0.40	1.18 (0.239)	3.69 ± 0.50	−0.35 (0.729)	3.79 ± 0.64	1.11 (0.271)	4.05 ± 0.43	0.889 (0.347)
	Master’s degree or higher	3.70 ± 0.52		3.76 ± 0.62		3.52 ± 0.72		4.21 ± 0.73	
Marital status	None	3.89 ± 0.42	1.15 (0.251)	3.68 ± 0.51	−0.46 (0.646)	3.76 ± 0.64	−1.186 (0.237)	4.01 ± 0.43	−2.93 (0.004)
	Yes	3.81 ± 0.37		3.72 ± 0.47		3.89 ± 0.66		4.24 ± 0.47	
Religion	Yes	3.89 ± 0.36	0.52 (0.603)	3.69 ± 0.46	−0.04 (0.969)	3.77 ± 0.56	−0.26 (0.792)	4.03 ± 0.45	−0.52 (0.603)
	No	3.86 ± 0.44		3.69 ± 0.52		3.79 ± 0.69		4.07 ± 0.44	
Clinical experience (years)	6 m–<1 year ^a^	3.83 ± 0.42	1.06 (0.368)	3.70 ± 0.57	0.37 (0.773)	3.83 ± 0.67	0.44 (0.722)	3.78 ± 0.40	5.36 (<0.001) a, b < d
	1–<3 ^b^	3.92 ± 0.38		3.73 ± 0.45		3.78 ± 0.61		3.94 ± 0.37	
	3–<5 ^c^	3.93 ± 0.41		3.63 ± 0.57		3.69 ± 0.69		4.04 ±.042	
	≥5 ^d^	3.82 ± 0.42		3.69 ± 0.49		3.83 ± 0.65		4.18 ± 0.44	
Working pattern	Three shifts	3.87 ± 0.41		3.67 ± 0.50		3.78 ± 0.65		4.05 ± 0.44	
Working department	Medical ward ^a^	3.88 ± 0.36	2.05 (0.132)	3.71 ± 0.47	2.51 (0.084)	3.72 ± 0.62	3.14 (0.046) a < c	4.04 ± 0.40	1.18 (0.308)
	Surgical ward ^b^	3.83 ± 0.47		3.63 ± 0.52		3.81 ± 0.68		4.05 ± 0.50	
	Other departments ^c^	4.12 ± 0.32		4.02 ± 0.51		4.27 ± 0.45		4.27 ± 0.31	
Monthly income (1000 won)	<2990 ^a^	3.77 ± 0.42	1.82 (0.165)	3.61 ± 0.45	5.58 (0.004) a, b < c	3.57 ± 0.85	2.33 (0.100)	4.00 ± 0.09	4.20 (0.016) a, b < c
	3000–3990 ^b^	3.88 ± 0.39		3.66 ± 0.49		3.79 ± 0.60		4.03 ± 0.41	
	≥4000	4.01 ± 0.50		4.05 ± 0.53		3.99 ± 0.62		4.34 ± 0.60	
Salary satisfaction	Satisfied	3.89 ± 0.51	1.22 (0.298)	3.83 ± 0.52	2.73 (0.068)	4.16 ± 0.45	6.32 (0.002) a > b, c	4.05 ± 0.45	0.34 (0.715)
	Neutral	3.91 ± 0.39		3.72 ± 0.50		3.72 ± 0.67		4.08 ± 0.46	
	Dissatisfied	3.81 ± 0.39		3.59 ± 0.47		3.71 ± 0.64		4.02 ± 0.42	
Department satisfaction	Satisfied ^a^	3.95 ± 0.38	5.06 (0.007) a > c	3.80 ± 0.49	7.74 (0.001) a > b, c	4.03 ± 0.53	23.32 (<0.001) a > b, c	4.09 ± 0.42	1.52 (0.222)
	Neutral ^b^	3.80 ± 0.43		3.59 ± 0.50		3.05 ± 0.66		4.04 ± 0.51	
	Dissatisfied ^c^	3.69 ± 0.36		3.40 ± 0.38		3.32 ± 0.57		3.90 ± 0.25	
Job satisfaction	Satisfied ^a^	4.00 ± 0.40	7.77 (0.001) a > b, c	3.87 ± 0.49	15.11 (<0.001) a > b, c	4.06 ± 0.58	16.28 (<0.001) a > b, c	4.15 ± 0.41	3.80 (0.024) a > c
	Neutral ^b^	3.81 ± 0.40		3.63 ± 0.45		3.63 ± 0.60		4.00 ± 0.49	
	Dissatisfied ^c^	3.68 ± 0.36		3.31 ± 0.43		3.41 ±.65		3.93 ± 0.30	

M, mean; SD, standard deviation. Scheffé post hoc test: a < b, c. Different superscript letters indicate statistically significant differences among groups (*p* < 0.05).

**Table 4:** Correlation between communication skills, grit, LMX, and nursing performance (N = 190).

Variables	Communication Skills	Grit	LMX	Nursing Work Performance
	r (*p*)			
Communication skills	1			
Grit	0.601 **	1		
LMX	0.274 **	0.485 **	1	
Nursing work performance	0.587 **	0.499 **	0.200 **	1

** *p* < 0.001.

**Table 5:** Association of communication skills, grit, and LMX to nursing performance in hierarchical linear regression (N = 190).

	Model I			Model II			Model III			Model IV		
Variables	β	t	*p*	β	t	*p*	β	t	*p*	β	t	*p*
Intercept		82.99	0.000		18.99	0.000		10.71	0.000		4.69	0.000
Age (31–40 years)	0.13	1.33	0.186	0.12	1.28	0.201	0.11	1.36	0.175	0.12	1.62	0.108
Marital status (yes)	0.07	0.76	0.448	0.06	0.68	0.499	0.09	1.19	0.235	0.12	1.86	0.065
Clinical experience (≥5 years)	0.08	0.76	0.449	0.08	0.82	0.416	0.14	1.50	0.136	0.18	2.31	0.022
Monthly income (≥4000 won)	0.10	1.28	0.203	0.10	1.23	0.220	−0.02	−0.35	0.730	−0.03	−0.53	0.596
Job satisfaction (satisfied)	0.16	2.22	0.027	0.11	1.45	0.148	0.04	0.55	0.585	−0.01	−0.24	0.812
LMX				0.13	1.76	0.081	−0.10	−1.32	0.187	−0.08	−1.21	0.228
Grit							0.54	7.42	0.000	0.23	3.14	0.002
Communication skills										0.52	7.67	0.000
Durbin–Watson										2.144		
F (*p*)	4.74			4.51 ***			12.88 ***			22.20 ***		
R^2^	0.114			0.129			0.331			0.495		
Adj R^2^ (change in adjusted R^2^)	0.090			0.100 (0.010)			0.305 (0.205 ***)			0.473 (0.168 ***)		

*** *p* < 0.001.

## Data Availability

Data can be shared with other researchers and journal editors upon request and for purposes of verifying results.

## Public Involvement Statement

No public involvement in any aspect of this research.

## Guidelines and Standards Statement

This manuscript was prepared in accordance with the Strengthening the Reporting of Observational Studies in Epidemiology (STROBE) guidelines for cross-sectional studies.
